# Discrimination of Free-Range and Caged Eggs by Chemometrics Analysis of the Elemental Profiles of Eggshell

**DOI:** 10.1155/2023/1271409

**Published:** 2023-02-28

**Authors:** Shunping Xie, Chengying Hai, Song He, Huanhuan Lu, Lu Xu, Haiyan Fu

**Affiliations:** ^1^Technology Center, China Tobacco Guizhou Industrial Co., Ltd., Guiyang 550009, Guizhou, China; ^2^The Modernization Engineering Technology Research Center of Ethnic Minority Medicine of Hubei Province, College of Pharmacy, South-Central Minzu University, Wuhan 430074, China; ^3^College of Material and Chemical Engineering, Tongren University, Tongren 554300, Guizhou, China

## Abstract

As one of the foods commonly eaten all over the world, eggs have attracted more and more attention for their quality and price. A method based on elemental profiles and chemometrics to discriminate between free-range and caged eggs was established. Free-range (*n*1 = 127) and caged (*n*2 = 122) eggs were collected from different producing areas in China. The content of 16 elements (Zn, Pb, Cd, Co, Ni, Fe, Mn, Cr, Mg, Cu, Se, Ca, Al, Sr, Na, and K) in the eggshell was determined using a inductively coupled plasma atomic emission spectrometer (ICP-AES). Outlier diagnosis is performed by robust Stahel–Donoho estimation (SDE) and the Kennard and Stone (K-S) algorithm for training and test set partitioning. Partial least squares discriminant analysis (PLS-DA) and least squares support vector machine (LS-SVM) were used for classification of the two types of eggs. As a result, Cd, Mn, Mg, Se, and K make an important contribution to the classification of free-range and caged eggs. By combining column-wise and row-wise rescaling of the elemental data, the sensitivity, specificity, and accuracy were 91.9%, 91.1%, and 92.7% for PLS-DA, while the results of LS-SVM were 95.3%, 95.6%, and 95.1%, respectively. The result indicates that chemometrics analysis of the elemental profiles of eggshells could provide a useful and effective method to discriminate between free-range and caged eggs.

## 1. Introduction

Chicken eggs are one of the main foodstuffs consumed worldwide, mainly consisting of eggshell, shell membrane, egg white, and yolk [1, 2]. It contains a wide range of nutrients and health-promoting components, such as lecithin, calcium ions, iron ions, and vitamin A [3–5]. Eggs have a high digestibility and absorption rate and are an inexpensive and abundant source of high-quality animal protein (about 13 grams of protein per 100 grams of egg) [6–8].

At present, various types of eggs exist in the Chinese market, such as organic and ordinary eggs, which have different nutritional composition and commercial value [9–11]. It has been demonstrated that the nutritional composition of eggs produced by hens fed different feeding methods (free-range or cage) and feeds (plant or animal sources) varies significantly [12, 13]. Free-range eggs contain about one-third and one-quarter less cholesterol and saturated fat than regular caged eggs, respectively, and there are significant differences in vitamin A, vitamin E, and beta-carotene content between free-range and caged eggs [14–16]. In addition, free-range eggs are considered to have higher nutritional value, flavor, and safety than caged eggs, so people are willing to pay a higher price for them. In recent years, egg adulteration and fraud have become more frequent, with traders selling caged eggs as free-range eggs for profit, which seriously undermines the lives, health, and legal rights of consumers. Therefore, it is important to develop a quick and reliable method to identify free-range or caged eggs on the market.

Presently, the methods applied to egg identification include high performance liquid chromatography [17, 18], gas chromatography-mass spectrometry [19, 20], hyperspectral imaging [21], elemental analysis [22], and near infrared spectroscopy [23, 24]. For example, Mi et al. [22] investigated the differentiation of Deqingyuan, Taihe, and crossbred eggs based on multielemental and lipidomic data combined with chemometric analysis and obtained a panel of 22 potential lipid markers for differentiating Deqingyuan, Taihe, and crossbred egg yolks. Rogers et al. [25] successfully used stable isotopes to analyse and discriminate between eggs produced under cage, barn, free-range, and organic farming systems in the Netherlands and New Zealand. However, fewer studies have been performed to discriminate between caged and free-range eggs in China. So, it is necessary to analyse and discriminate between caged and free-range eggs in China.

In China, a significant difference between caged and free-range eggs is the use of different feeds. Free-range eggs are produced in a small scale by individual farmers using grains as the main feed, while caged eggs are produced in a large scale using commercial feeds [26, 27]. The differences in feeding styles can cause the differences in elemental contents, which can be used to identify different types of eggs [28, 29]. Therefore, the aim of this work is to develop an egg classification method to discriminate between free-range and cage eggs using element analysis combined with chemometrics. In this work, using egg shells as an analytical object to distinguish between free-range and caged eggs, an inductively coupled plasma-atomic emission spectrometer (ICP-AES) was used to analyse the content of 16 mineral elements in eggshells. Various classification models such as PLS-DA and LS-SVM were established to discriminate between free-range and caged eggs, and the performance of different methods was compared to obtain the best classification model.

## 2. Materials and Methods

### 2.1. Experimental Materials and Reagents

Representative caged and free-range egg samples were collected from different producing areas in China. 127 free-range samples and 122 caged eggs were analysed. All egg samples are purchased directly from the manufacturer after confirming the type in 2019. Five eggs will be taken from each batch of samples for parallel analysis, and the remaining two eggs will be used as spares. The detailed information concerning the samples is shown in Table 1.

Standard reserve solutions (1000 *μ*g·mL^−1^) of Zn, Cd, Co, Cr, Cu, Ca, Mg, Mn, Mo, Ni, Pb, Sr, Fe, Na, and K were obtained from the National Standard Material Center of China. HNO_3_ and H_2_O_2_ were purchased from Sinopharm Chemical Reagent Co., Ltd.

### 2.2. Digestion of Eggshells

The eggshell was rinsed with tap water after removing the internal membrane. Then, the eggshell was washed with deionized water and dried at 120°C using electric sleeve heating. About 1 gram of dried eggshell was weighed accurately on the electro-optic balance, then smashed into small pieces, and put into a 50-mL conical flask. For digestion, 8 mL HNO_3_ (65%, w/w %) and 2 mL H_2_O_2_ (30%, w/w %) were added. The conical flask was heated and kept at 60°C until a colourless solution was obtained. The solution was cooled naturally and transferred to a 50 mL volumetric flask, where deionized water was added to a constant volume. The blank was prepared using 4 mL HNO_3_ (65%, w/w %) and 1 mL H_2_O_2_ (30%, w/w %).

### 2.3. Elemental Analysis by ICP-AES

The concentration of the 16 mineral elements in the eggshells was determined using a Shimadzu ICPS-7510 sequential plasma emission spectrometer (Shimadzu, Kyoto, Japan). The spectrometer parameters were as follows: power: 1300 W; plasma flow rate: 15 L min^−1^; carrier gas flow rate: 0.8 L min^−1^; auxiliary flow rate: 0.2 L min^−1^; atomization flow rate: 0.8 L min^−1^; pump flow rate: 1.5 mL min^−1^; axial observation distance: 15 mm; and the instrumentation stabilization time of 30 s. Analytical lines (Table 2) were selected by considering the overlapping and intensity of signals. A standard curve was developed for each element. For each batch, elemental contents were reported as the average of eggshell samples analysed in triple.

### 2.4. Data Preprocessing, Outlier Diagnosis, and Data Splitting

All data preprocessing and further analysis were performed using Matlab 7.0.1 (Mathworks, Sherborn, MA). When the measured data are influenced by significant bias and other undesirable factors, the performance and reliability of classification modeling would be degraded; therefore, the potential outliers should be detected and removed. In order to solve the masking effect of multiple outliers, the Stahel–Donoho estimate (SDE) of outlyingness was used for outlier diagnosis of elemental data, which is a robust statistical method with dimension reduction techniques [30]. The SDE calculates a large number of projections of randomly selected objects in each direction, and through the robust positioning and scatter estimators of the projection, the SDE outlier of each sample is obtained. In this work, the SDE was used for outlier diagnosis in free-range and caged eggs separately.

Subsequently, the measured data are divided into a training set and a prediction set by the Kennard and Stone (K-S) algorithm [31]. The K-S algorithm will select a representative training set to make the objects as scattered in the data space as possible. Because the distributions of two classes of eggs were not the same, the K-S method was performed separately for the free-range and caged eggs.

### 2.5. Multivariate Discriminate Analysis

For pattern recognition, linear partial least squares discriminant analysis (PLS-DA) [32] and nonlinear least squares support vector machine (LS-SVM) [33] are performed to distinguish free-range and caged eggs. Monte Carlo Cross Validation (MCCV) [34] is used to evaluate the number of PLS-DA latent variables, and the parameters of LS-SVM are optimized to obtain the lowest MCCV error rate (MCCVER) and reduce the risk of model overfitting.

Principal component analysis (PCA) is an unsupervised data dimensionality reduction method, which converts a set of potentially correlated variable data into a set of linearly uncorrelated variables through orthogonal transformation, and the converted variables are called principal components. In recent years, PCA has been widely used for classification and identification of varieties, origins, and adulteration of food and agricultural products [35]. Partial least squares discriminant analysis (PLS-DA) is a supervised discriminant analysis statistical method which is often used to deal with classification and discriminant problems. It can well solve those classification problems in which the differences between groups are small and the sample sizes of the groups vary widely [36]. LS-SVM (least squares support vector machines) is mainly used to solve pattern classification and function estimation problems. The optimization of the model parameters such as the kernel function parameter (*σ*) and the regularization parameter (*γ*) is required when using it. The kernel parameter has a direct impact on the complexity of the distribution of low-dimensional sample data in the mapping space, while the regularization parameter is related to the fit of the model to the training samples and the generalization ability of the model [37].

Sensitivity and specificity were used to estimate and compare the performance of classification models. Free-range eggs are denoted as “positives,” and caged eggs are denoted as “negatives.” Sensitivity (Sens), specificity (Spec), and overall accuracy (Accu) can be computed as follows:

(1) $$\begin{array}{c} Sens=\frac{TP}{TP+FN}\text{,}\\ Spec=\frac{TN}{TN+FP}\text{,}\\ Accu=\frac{TN+TP}{TN+TP+FN+FP}\text{.} \end{array}$$

Among them, TP represents true positive, FN represents false negative, TN represents true negative, and FP represents false positive.

## 3. Results and Discussion

### 3.1. Elemental Data of Eggshells

Table 3 showed the ICP-AES analysis results of 16 elements in free-range and caged eggs. The elemental contents of Ca, Mg, Na, and K were the highest in free-range and caged eggs. Among them, free-range eggs have higher content of Ca, Mg, and Se compared to caged eggs, while caged eggs have higher content of Na, K, Al, Sr, Fe, and Mn, which is consistent with previous studies [38]. It is noteworthy that caged eggs have higher content of heavy metals such as Pb, Cd, Cr, and Cu, and there is no detected Cd element in the free-range eggs. It is known that elements Ca, Mg, Na, and K are involved in various metabolisms in the human body and are essential elements required by the human body, and Se is an important nutrient for the prevention of tumors and liver diseases as well as the improvement of immunity.

**Figure 1:**
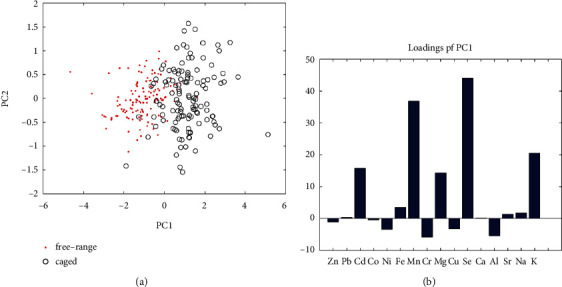
(a) Principal component 1 (PC1) and principal component 2 (PC2) score plot in free-range and caged eggshells. (b) PC1 loadings of the elemental profiles of free-range and caged eggshells.

To illustrate the data distribution, principal component analysis (PCA) was used on the column-wise and row-wise rescaled data without outlier diagnosis (Figure 1). Principal component 1 and principal component 2 explained 90.06% of all data variation, and projection of the raw data onto PC1 and PC2 to obtain score plots showed that free-range eggs and caged eggs basically achieved a better separation, which were clustered into two groups, respectively, where some samples overlapped due to the small differences in trace element contents in these samples (Figure 1(a)). The loading plot of principal component 1 is shown in Figure 1(b), which shows that the contents of Cd, Mn, Mg, Se, and K contribute significantly to the separation between groups achieved by PC1, while the elements Zn, Co, Ni, Gr, Cu, and Al have negative effects in the classification. The combined content analysis showed that Cd, Mn, Mg, Se, and K had important contributions in the classification of free-range eggs and caged eggs and could be used as effective elements to distinguish free-range eggs from caged eggs. Although the PCA model achieved the distinction between free-range eggs and caged eggs, the classification accuracy did not reach 100%. So, supervised chemometric models are still needed to achieve accurate classification of the two classes.

### 3.2. Development of Classification Models

**Figure 2:**
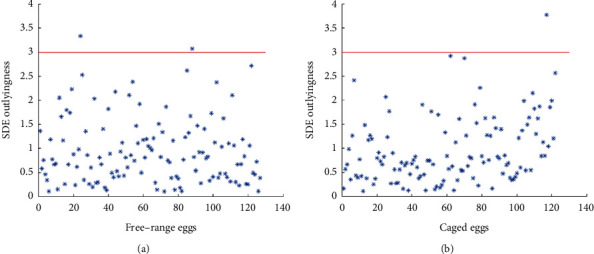
SDE outlier diagnosis of 127 free-range eggshells (a) and 122 caged eggshells (b) based on rescaled element data.

Considering the relative contents of different elements and the difference in each sample weight, rescaling of the data was necessary to analyse the elemental data. In this work, the data for an object was divided by its sample weight followed by a column-wise transformation into unit variance for each element. The SDE outlyingness analysis was performed separately on each of the two classes using the rescaled data. Outlying values were estimated by 1,000 random projections.  Figure 2 shows the SDE outlier diagnostic curve for 127 free-range eggshells and 122 caged eggshells, according to the 3-*σ* rule. A critical value of 3 was adopted, and an object with an outlyingness value above 3 was considered an outlier. 2 and 1 objects for free-range and caged eggs were detected as outliers, respectively (Figure 2). Further tracing of the samples indicates that the labels of these eggs were suspicious. Therefore, these objects were excluded from discriminant analysis.

After eliminating outliers, the remaining 125 free-range eggs and 121 caged eggs were used to develop and test classification models. The K-S algorithm was performed separately for the two groups, dividing the free-range eggs into 80 training subjects and 45 test subjects and then dividing the caged eggs into 80 training subjects and 41 test subjects. Therefore, a training set of 160 (80 + 80) objects and a test set of 86 (45 + 41) objects were obtained to develop and evaluate the classification model.

**Figure 3:**
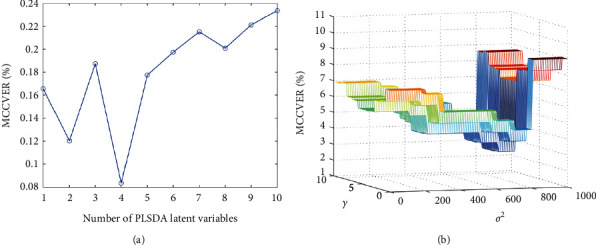
MCCV optimization of PLS-DA (a) and LS-SVM (b) model parameters.

The PLS-DA model and the LS-SVM model based on the eggshell element data were established. The two parameters *γ* and *σ* are optimized in the LS-SVM model. The kernel width parameter *σ* is related to the data confidence and the nonlinear nature of the model, and the smaller *σ* means the narrower the kernel width, which may force the model to shift to more complex nonlinear solutions. Another parameter *γ* is a regularization parameter, which involves the trade-off between learning accuracy and structural risk. To simultaneously optimize (*σ*, *γ*), a grid search method was performed by MCCV. In addition, MCCV is to estimate the number of meaningful PLS-DA latent variables (LV). All parameters of PLS-DA and LS-SVM are optimized by minimizing the MCCV error rate. For MCCV, 70% of the samples were used for the training set and 30% for the test set. The random data split number of MCCV is 100, and the optimization of model parameters is shown in Figure 3.

**Figure 4:**
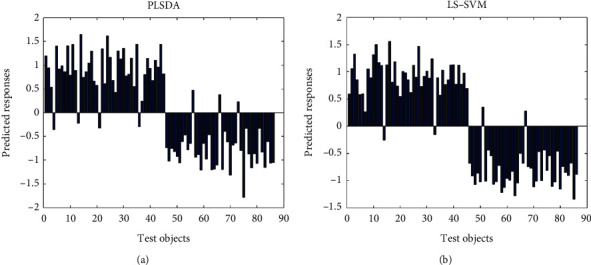
Classification results by (a) PLS-DA and (b) LS-SVM. Objects 1–45, free-range eggs; objects 46–86, caged eggs.

The optimization parameters and classification results of PLS-DA and LS-SVM models are shown in Table 4. For PLS-DA, the model has the lowest MCCVER (8.36%) when LV = 4 (Figure 3(a)), which indicates that better classification of free-range eggs and caged eggs can be achieved with lower model complexity. For LS-SVM, the lowest value of MCCVER (2.47%) was obtained when the values of *σ* and *γ* were 700 and 5, respectively; so, this parameter was chosen for classification.  Figure 4 shows the score plot of the prediction set of the PLS-DA model (Figure 4(a)), which shows that four free-range eggs were misclassified as caged eggs and three caged eggs were misclassified as free-range eggs, and the models' accuracy, sensitivity, and specificity were 91.9%, 91.1%, and 92.7%, respectively. In the LS-SVM model, 2 free-range eggs were misclassified as caged eggs and 2 caged eggs were misclassified as free-range eggs, with the models' accuracy, sensitivity, and specificity of 95.3%, 95.6%, and 95.1%, respectively. The LS-SVM model has higher classification accuracy compared to PLS-DA, demonstrating that LS-SVM is more suitable for the classification of free-range eggs and caged eggs. According to previous studies, the discrimination of free-range, caged, organic, and ordinary eggs is mainly based on the analysis of chemical components such as carotenoids [39], lipid extracts [40], proteins, and moisture in eggs [41], which enable an accurate identification of different varieties of eggs, but the pretreatment of these methods is more complicated. In addition, mineral element-based methods combined with chemometrics have been successfully applied to identify free-range and caged eggs. In Dao's study, significantly higher levels of the mineral elements P, Mg, and Na and lower levels of the trace elements Cu, Fe, K, S, and Mn were found in Australian free-range eggs, and a good classification of free-range and caged eggs from Australia and Syria was achieved [38]. The above studies show that mineral element-based methods combined with chemometrics can achieve accurate identification of free-range eggs and caged eggs in China.

## 4. Conclusions

As a result, 16 mineral elements (Zn, Pb, Cd, Co, Ni, Fe, Mn, Cr, Mg, Cu, Se, Ca, Al, Sr, Na, and K) in eggshells combined with chemometrics can distinguish between free-range and caged egg samples, and Cd, Mn, Mg, Se, and K have a significant influence on the classification as potential factors for free-range and caged eggs. PCA, PLS-DA, and LS-SVM are applied to the classification of free-range and cage-reared eggs. Both PLS-DA and LS-SVM could obtain good discrimination results. Especially, LS-SVM can obtain better classification performance with an overall accuracy of 95.3%, a sensitivity of 95.6%, and a specificity of 95.1%. So elemental analysis combined with chemometrics can be used as a simple and effective method to identify free-range and caged egg samples.

## Figures and Tables

**Table 1:** Detailed information of the free-range and caged eggs analysed.

Producing area	Batch size	Type^*a*^
Guizhou	10	*F*
Guizhou	11	*C*
Henan	22	*F*
Henan	18	*C*
Anhui	20	*F*
Anhui	16	*C*
Jiangsu	15	*F*
Jiangsu	20	*C*
Hubei	26	*F*
Hubei	19	*C*
Guangxi	16	*F*
Guangxi	17	*C*
Hunan	18	*F*
Hunan	21	*C*

^*a*^ *F* = “free-range” and *C* = “caged”.

**Table 2:** Analysis of wavelengths of 16 mineral elements in free-range and caged eggshells.

Element	Wavelength (nm)	Element	Wavelength (nm)
Zn	213.85	Pb	220.35
Cd	226.50	Co	228.61
Ni	231.60	Fe	238.20
Mn	257.61	Cr	267.71
Mg	280.26	Cu	324.75
Se	361.38	Ca	393.36
Al	396.15	Sr	407.77
Na	589.60	K	766.49

**Table 3:** Analytical results of 16 elements in free-range and caged eggshells.

Elements	Average levels (SD) (*μ*g/g)		DL^*a*^ (*μ*g/g)
	Free-range	Caged	
Zn	1.72 (0.39)	2.33 (0.51)	0.05
Pb	1.04 (0.27)	2.16 (0.19)	0.09
Cd	0^*b*^	0.34 (0.08)	0.03
Co	0.22 (0.04)	0.32 (0.09)	0.03
Ni	0.44 (0.12)	0.59 (0.10)	0.05
Fe	2.1 (0.6)	3.2 (1.0)	0.04
Mn	0.67 (0.13)	1.01 (0.31)	0.07
Cr	0.71 (0.28)	2.32 (1.03)	0.02
Mg	6042 (2106)	5363 (1849)	0.08
Cu	1.56 (0.31)	2.13 (0.55)	0.07
Se	4.22 (1.68)	2.18 (1.01)	0.04
Ca	366310 (46778)	342560 (35458)	0.08
Al	71.9 (6.5)	88.5 (11.7)	0.10
Sr	120.4 (24.8)	171.3 (44.6)	0.02
Na	2156 (388)	3016 (612)	0.33
K	1850 (302)	2305 (226)	0.76

^*a*^The detection limit was related to the 3*σ* signal, where *σ* was estimated from 11 repeated measurements of the blank. ^*b*^Nondetected.

**Table 4:** Classification of free-range and caged eggs using rescaled elemental data of eggshells by PLS-DA and LS-SVM.

Models	Parameters	ERMCCV (%)	Accuracy (%)	Sensitivity (%)	Specificity (%)
PLS-DA	4^*a*^	8.36	91.9	91.1 (41/45)^*b*^	92.7 (38/41)^*c*^
LS-SVM	(700, 5)^*d*^	2.47	95.3	95.6 (43/45)	95.1 (39/41)

^*a*^Number of PLS-DA latent variables. ^*b*^TP/TP + FN. ^*c*^TN/TN + FP. ^*d*^Values of (*σ*^2^, *γ*).

## Data Availability

The data supporting the findings of the current study are available from the corresponding author upon request.

## Abbreviations

ICP-AES: Inductively coupled plasma atomic emission spectrometer
PLS-DA: Partial least squares discriminant analysis
LS-SVM: Least squares support vector machines
SDE: Stahel–Donoho estimation
PCA: Principal component analysis
SDE: Stahel–Donoho estimate
MCCV: Monte Carlo cross validation
ERMCCV: Misclassification rate of MCCV
LVs: Latent variables
SD: Standard deviations.
